# PHQ-9, CES-D, health insurance data—who is identified with depression? A Population-based study in persons with diabetes

**DOI:** 10.1186/s13098-023-01028-7

**Published:** 2023-03-22

**Authors:** Ute Linnenkamp, Veronika Gontscharuk, Katherine Ogurtsova, Manuela Brüne, Nadezda Chernyak, Tatjana Kvitkina, Werner Arend, Imke Schmitz-Losem, Johannes Kruse, Norbert Hermanns, Bernd Kulzer, Silvia M. A. A. Evers, Mickaël Hiligsmann, Barbara Hoffmann, Andrea Icks, Silke Andrich

**Affiliations:** 1 Institute for Health Services Research and Health Economics, German Diabetes Center, Leibniz Center for Diabetes Research at Heinrich-Heine-University Düsseldorf, Düsseldorf, Germany; 2 German Center for Diabetes Research, Partner Düsseldorf, München-Neuherberg, Germany; 3 Department of Health Services Research, CAPHRI Care and Public Health Research Institute, Maastricht University, Maastricht, the Netherlands; 4 Institute for Health Services Research and Health Economics, Centre for Health and Society, Medical Faculty and University Hospital Düsseldorf, Heinrich-Heine-University Düsseldorf, Düsseldorf, Germany; 5 pronova BKK, statutory health insurance, Ludwigshafen, 67058 Germany; 6 Clinic for Psychosomatic and Psychotherapy, University Clinic Gießen, Gießen, Germany; 7 Research Institute Diabetes Academy Mergentheim (FIDAM), Bad Mergentheim, Germany; 8 Department of Clinical Psychology and Psychotherapy, University of Bamberg, Bamberg, Germany; 9 Trimbos Institute, Netherlands Institute of Mental Health and Addiction, Utrecht, the Netherlands; 10 Institute for Occupational, Social and Environmental Medicine, Centre for Health and Society, Faculty of Medicine, Heinrich-Heine University Düsseldorf, Düsseldorf, Germany

**Keywords:** Depressive disorder diagnosis, Depressive disorder epidemiology, Diabetes Mellitus Type 2 psychology, Diabetes complications

## Abstract

**Aims:**

Several instruments are used to identify depression among patients with diabetes and have been compared for their test criteria, but, not for the overlaps and differences, for example, in the sociodemographic and clinical characteristics of the individuals identified with different instruments.

**Methods:**

We conducted a cross-sectional survey among a random sample of a statutory health insurance (SHI) (n = 1,579) with diabetes and linked it with longitudinal SHI data. Depression symptoms were identified using either the Centre for Epidemiological Studies Depression (CES-D) scale or the Patient Health Questionnaire-9 (PHQ-9), and a depressive disorder was identified with a diagnosis in SHI data, resulting in 8 possible groups. Groups were compared using a multinomial logistic model.

**Results:**

In total 33·0% of our analysis sample were identified with depression by at least one method. 5·0% were identified with depression by all methods. Multinomial logistic analysis showed that identification through SHI data only compared to the group with no depression was associated with gender (women). Identification through at least SHI data was associated with taking antidepressants and previous depression. Health related quality of life, especially the mental summary score was associated with depression but not when identified through SHI data only.

**Conclusion:**

The methods overlapped less than expected. We did not find a clear pattern between methods used and characteristics of individuals identified. However, we found first indications that the choice of method is related to specific underlying characteristics in the identified population. These findings need to be confirmed by further studies with larger study samples.

**Supplementary Information:**

The online version contains supplementary material available at 10.1186/s13098-023-01028-7.

## Key points

- Patients with diabetes often have comorbid depression. Those patients are struggling to meet their treatment goals. Thus, they have a higher risk of getting diabetes related complications as for example coronary heart diseases.
- A lot of different tools and instruments are available to diagnose depression, to screen for depression among patients with diabetes or to identify depression symptoms or depressive disorder in clinical or epidemiological studies, including interview, questionnaires or claims data. It would be helpful to know if the tools that are used identify the same people or, if this is not the case, whether people identified by different tools have different characteristics or health outcomes.
- We found that different methods do not identify the same people with depression. There was no clear pattern of differences between the identified groups, however, we found some initial indications that the method chosen is related to particular underlying characteristics in the population identified. Further research with larger data sets is necessary to see if there are differences among the persons that are identified by different tools to give recommendations which screening tool to use for what purpose.

**Supplementary Information:**

The online version contains supplementary material available at 10.1186/s13098-023-01028-7.

## Introduction

Patients with diabetes have an increased prevalence of depression compared to the general population [1]. Although it remains controversial if diabetes leads to depression or vice versa or if there is a bidirectional association, there is sufficient evidence that depression can have a serious impact on a person’s wellbeing and their ability to self-manage their diabetes [2–5]. Individuals with diabetes and comorbid depression are found to have unfavorable diabetes related outcomes such as a reduced adherence to their diabetes treatment, higher HbA1c levels, increased diabetes symptoms, or unfavorable micro-, and macrovascular outcomes [2–6]. Beyond unfavorable health outcomes, Brüne et al. (2021) found that people with diabetes and depression had almost two times higher total health care cost compared to people with diabetes without depression [7]. Despite the relevance of comorbid depression in people with diabetes, it is assumed that only 50% are recognized and an even smaller amount is appropriately treated [2].

Several methods are used to identify depression or to estimate the prevalence of it. Prevalence estimates of depression among people with diabetes differ, which is also due to the fact that a range of different methods are used to assess depression [1, 8]. Three systematic reviews found, that in studies where a questionnaire was used to assess depression, the prevalence was about two to three times higher than in those that used a diagnostic interview [8–10].

The method used to assess the presence of depression depends on several factors. For example, it may depend on study design, time constraints, personal preferences of the researchers, availability or the aim of the assessment. Furthermore, there are a variety of questionnaires, each with a different objective and somewhat different background or focus [11–14]. Knowledge of the different methods and instruments to assess depression is therefore important. Up to now, there are a number of studies available that validate these questionnaires in general [15, 16]. Very few studies have compared the different instruments for identifying depression among patients with diabetes. These studies either intended to validate a certain instrument against another in a specific population or wanted to compare psychometric properties or internal reliability [17–20].

A method other than questionnaires is the use of diagnosis in statutory health insurance (SHI) data to identify persons with depressive disorder. Up to now, there is no study, in which SHI data was used for comparison purposes. In our study, we used two of the most common instruments in addition to SHI data to investigate whether the different methods identify - more or less - the same individuals or whether they identify different individuals. In particular - if the identified individuals differ - we were interested in possible patterns of characteristics of the identified groups. Thus, in contrast to existing validation studies, the aim of this study was to assess and describe in detail the overlap and the differences between groups identified by different methods to find persons with depression (symptoms or disorders), as well as potential associations between individual and clinical characteristics and the method used to identify a person.

Specifically, three methods to identify depression were used and compared: the Centre for Epidemiological Studies Depression (CES-D) scale, the Patient Health Questionnaire-9 (PHQ-9) - the two most frequently evaluated questionnaires among people with diabetes [20] - or a diagnosis in SHI data. In this way, we aimed to gain basic insights and better understand the issues associated with the use of different methods.

## Methods

### Study design

The study design and recruitment of participants have been described elsewhere [21]. In brief, a cross-sectional survey was conducted in a random sample of individuals with diabetes (N = 4,053) insured by one SHI covering 673,366 persons in Germany. Individuals with diabetes type 1 or 2 were identified using an algorithm taking into account diagnosis based on the 10th International Classification of Diseases (ICD-10) for ‘diabetes’ (E10–E14), prescription of antihyperglycemic drugs (Anatomical-Therapeutic-Chemical [ATC] classification A10), and documentation of blood glucose, or a HbA1c measurements. This algorithm has been validated and used in previous studies [22]. We linked data of the survey to longitudinal SHI data on an individual level. The initial aim of the study was to assess differences in people with diabetes and with and without depression regarding costs and health related quality of life. The presented analyses are secondary analyses that were developed in the course of the study.

### Data source

The baseline survey was a 9-page postal questionnaire conducted in 2013. It assessed information on sociodemographic characteristics such as age, sex, and years of education, duration of diabetes, and type of diabetes. PHQ-9 and the German version of the CES-D were used to assess depression symptoms.

SHI data on health care utilization patterns and health care costs for all in- and outpatient treatments were available for the period covering four quarters before and after the quarter of the baseline survey.

### Study population

Of 46,566 individuals with diabetes in the SHI 3,642 persons were randomly selected and contacted to participate in the study. In total 1,860 persons sent back their questionnaire (response rate: 51%) and gave written informed consent to use their SHI data. Responders did not differ from the non-responders in having a history of depression diagnosis [23]. For 201 of these persons, a lack of data over the complete observation period existed, e.g. because the person switched health insurance during that time. In total 1,659 persons were considered for the analysis. Further 80 persons were excluded as they provided incomplete information in the questionnaire. Thus, a total of 1,579 persons were included in our analysis (Appendix Fig. 1).

Ethical approval was obtained from the ethics committee of the Heinrich Heine University Düsseldorf and is available under the study reference 3762.

### Main outcome – assessment of depression

#### CES-D

The CES-D and the German version of it (Allgemeine Depressionsskala) are brief self-report measures, designed to assess symptoms of depression in the general population in epidemiological studies among nine signs and symptoms of depression defined by the American Psychiatric Association Diagnostic and Statistical Manual, fourth edition [11]. Several studies have assessed the validity of the CES-D in different populations [24, 25]. We used the short form of the German version of the CES-D in our study (allgemeine Depressionsskala Kurzform (ADS-K)) [25]. The instrument comprises 15 statements regarding depression. Based on a four-point scale (ranging from “rarely or never” (0 point) to “frequently, all the time” (3 points)), the frequency of depressive symptoms occurring during the last week can be assessed. A score that can range from 0 to 45 is built by adding up the points from each statement. We used a cut-off value of ≥ 17 to define clinically meaningful depressive symptoms as suggested by validation studies [25].

#### PHQ-9

The PHQ-9 is a multipurpose instrument used to screen, monitor and measure the severity of depression symptoms. The PHQ-9 can be assessed using different methods: as a diagnostic algorithm to make a probable diagnosis of major depressive disorder using the nine criteria of the Diagnostic and Statistical Manual of Mental Disorders, Fourth Edition (DSM-IV) or to test for other depressive disorders and a cut-off based on summed-item scores to assess the severity of depression symptoms [12]. The algorithm is the scoring method that was originally proposed to screen for depression. Within this study we focused on the PHQ-9 as a screening instrument. According to Kroenke et al. (2001) we defined depression when two or more of the nine symptoms were present at least “more than half the days” in the past two weeks, and one of the symptoms was depressed mood or anhedonia. If the thought of suicide was present, it is considered to be present, regardless of the reported duration [12]. Several studies have used the PHQ-9 to assess depression among individuals with diabetes and used a similar approach [2, 26].

#### Depression in SHI data

For a diagnosis in SHI data a ICD-10 code for the diagnosis of unipolar depression during the study period of nine quarters was required. Diagnosis of unipolar depression included the following codes:

F32.0-F32.9 Depressive episode,

F33.0-F33.9 Recurrent depressive disorder,

F34.1 Dysthymia,

F38.1. Other recurrent mood [affective] disorders and.

F41.2 Mixed anxiety and depressive disorder.

#### Group composition based on depression measurement

We classified the participants into eight groups after linking SHI data with survey data. Group 1 reported depression symptoms in the CES-D and PHQ-9 and had a diagnosis in SHI data. Group 2 reported depression symptoms in the CES-D and PHQ-9 but had no diagnosis in SHI data. Group 3 had symptoms according to the PHQ-9 but not according to the CES-D and had a diagnosis in SHI data. For group 4 no symptoms were reported with the PHQ-9 but with the CES-D and they had a diagnosis in SHI data. Group 5 was only identified with the CES-D, Group 6 only with the PHQ-9 and group 7 only with a diagnosis in SHI data. Group 8 had no depression symptoms or diagnosis and was considered as a reference group (Appendix Table 1).

### Possible associated variables and covariates

All potentially associated variables and covariates considered as potential predictors were recorded during the baseline survey, except information on clinical and disease related measures (based on SHI data). Based on a literature review and clinical expertise, we considered socio-demographic variables, patient-reported measures on health-related quality of life (HRQoL) and diabetes related distress as well as clinical and disease-specific variables.

The following variables were included as sociodemographic factors: age, gender, marital status (married, single, divorced or separated, widowed), relationship status (with/without partner), origin (resident in Germany since birth/not residing in Germany since birth) as well as employment (yes/no), and retirement status (yes/no). The International Standard Classification of Education (ISCED) was used to categorize participants according to the duration of their education (< 10 years, 10–14 years, > 14 years) [27]. Furthermore, type and duration of diabetes were also assessed in the baseline survey as well as information on a previous diagnosis of depression by a health professional.

HRQoL was investigated using the 12-item Short Form health survey (SF-12), a multipurpose generic measure of health status [28]. The SF-12 can be used to compose a physical health and a mental health summary score (PCS-12 and MCS-12).

We also assessed diabetes-specific distress using the Problem Areas in Diabetes Scale (PAID), a 20-item scale consisting of emotional problems commonly reported among patients with diabetes [19].

SHI data was used to assess clinical and disease related measures. Comorbidities were measured using diagnostic groupings, which are necessary for the morbidity-oriented risk structure adjustment by SHIs in Germany. We used the number of coded morbidity groups in the year prior to the baseline survey (2012) to assess the number of comorbidities [29].

Healthcare costs were calculated from the perspective of a SHI including all costs imposed to the SHI. We took net costs into consideration without taking discounts into account. Costs were analyzed for every person individually, covering the survey quarter plus the four quarters before and after, a total of nine quarters.

The adapted Diabetes Complications Severity Index (aDCSI) was used to assess diabetes complications thereon to determine diabetes severity [30].

Treatment of diabetes was assessed by looking for prescription of insulin or oral antihyperglycemic drugs (OADs) in the SHI data for each participant during the course of the study. Additional it was checked whether persons took antidepressants during the course of the study. These were defined by the ATC Code N06A.

### Statistical analyses

We described the study population by using mean, standard deviation and median for quantitative variables as well as frequency and percentage for categorical variables. We used the Mann–Whitney U test for comparison of quantitative variables in two groups and Kruskal-Wallis test for three and more groups. Pearson’s chi-square test was conducted to assess if differences for categorical variables were significant. P-values related to the aforementioned tests show the probability to observe the actual value of the related test statistic or even more extreme values of it assuming the null hypothesis that there are no differences between groups. Smaller p-values indicate against the null hypothesis.

To compare the eight groups, we handled the missing data (cf. description of the study population and Table 1) with the machine learning based R-algorithm missForest to impute. To assess the quality of the imputation we calculated out-of-bag (OOB) imputation errors as the proportion of false classified cases (PFC) for categorical and as normalized root of mean squared error (NRMSE) for quantitative variables. Since the comparison of all eight groups to each other (the so called many-to-many problem) requires 28 pairwise comparisons, each with respect to a variety of characteristics, one should expect a considerable number of false rejections/effects. In order to be able on the one hand to control the type I error (i.e., rejection of at least one true null hypothesis, also known as family-wise error rate) and on the other hand to see any effects after multiple adjustment (done by the Bonferroni correction), we focused on the comparison of seven groups with depressive disorder to the group with no depression or depressive symptoms (i.e., group 8) as the reference group (the so called many-to-one problem). We used a multinomial logistic regression to model the group membership, whereby the log odds of being in one group relative to being in the reference group is modelled as a linear combination of predictor variables. Thus, an indirect comparison of seven groups with depressive disorder to each other may be done by comparing those differences to the reference group.

Gender, age, marital status, employment status, type of diabetes and diabetes duration, insulin and OAD usage, aDCSI score, previous depression and intake of antidepressant medication, number of comorbidities, HRQoL, PAID score, and total health care costs were used as potential candidates for independent variables in the multinomial model. We selected the finale multinomial model by keeping important variables (age, sex, comorbidities, MCS-12 and PCS-12), removing collinear variables as well as minimizing Akaike information criterion (AIC). The final model includes the independent variables: age, gender, taking insulin, previous depression, taking antidepressant, the number of comorbidities, HRQoL, and the PAID score.

P-values related to the estimates of the multinomial regression odds ratios (OR) for being in a group with depressive disorder compared to the reference group, are the probabilities to observe the actual value of the OR or more extreme values and under the null hypothesis that there is no effect (OR = 1). Smaller p-values are an indication, that null hypothesis may be wrong and there is an effect.

The significance level (also for multiple comparisons) was set to α = 0·05.

## Results

### Description of the study population

Table 1 describes the 1,579 participants and their characteristics. For 271 subjects in the total sample (17·2%) data of at least one variable in the baseline survey were missing while 1,308 persons had complete data.

Participants had a mean age of 67 years and almost 40% were female. About 90% were German and 84% were in a relationship. About one in five had more than 14 years of education. Almost 70% of the participants were retired. More than 75% were married, around 7% were divorced or separated and 12·4% were widowed.

On average participants had diabetes for 11 years, the majority had T2DM (85·9%). About one-third of the participants were treated with insulin, around 67% took OAD. 17·5% took antidepressants. The mean healthcare costs in our sample were 10,123€. Participants had on average 41·7 points on the physical component summary scale (PCS) of the SF-12 and 50·1 on the mental component summary scale (MCS). The average PAID Score in the sample was 19·4. 14% of people in the sample reported that they had previously been diagnosed with depression.

**Table 1:** Baseline characteristics of the DiaDec sample

	n (%), mean ± SD, median
Sample size, n	1,579
Age, years, n = 1579	67·0 **±** 9·9, 69·0
Sex, female, n = 1579	597 (37·8)
Origin, Germany n = 1577	1,397 (88·6)
Family status, in a relationship, n = 1556	1,306 (83·9)
Marital status, n = 1573	
Married	1,188 (75·5)
Divorce/separated	112 (7·1)
Widowed	195 (12·4)
Employment status, employed, n = 1547	402 (26·0)
Retirement status, retired, n = 1564	1,089 (69·6)
Level of education, ISCED ≥ 14 years, n = 1570	337 (21·5)
Diabetes duration in years, n = 1533	11·0 **±** 8·3, 9·0
Type of Diabetes, n = 1566	
Type 1 Diabetes	128 (8·2)
Type 2 Diabetes	1,345 (85·9)
Type unknown/other	93 (5·9)
Diabetes severity aDCSI, n = 1579	3·0 **±** 2·2, 3·0
Number of comorbidities, n = 1579	3·7 **±** 2·1, 3·0
Treatment, n = 1579	
Taking insulin	486 (30·8)
Taking oral antihyperglycemic drugs	1,071 (67·8)
Taking antidepressants	276 (17·5)
Health care costs for 2 years, €, n = 1579	10,123·0 **±** 13,188·2, 6,112·7
Health related Quality of Life^$^, n = 1544	
physical component summary scale of the SF-12 (PCS-12)	41·7 **±** 10·9, 43·5
mental component summary scale of the SF-12 (MCS-12)	50·1 **±** 10·5, 53·3
Problem Areas in Diabetes Scale (PAID)^∞^, n = 1512	19·4 ± 17·6, 14.0
Previous depression, n (%) (self-reported), n = 1575	
Yes	225 (14·3)
No	1,041 (66·1)
unknown	309 (19·6)

Percentages of categorical variables computed with respect to the total number of subjects within the sample

SD = standard deviation

^$^ range from 0 to 100, zero indicates the lowest level of health measured by the scales and 100 indicates the highest level of health

^∞^ Possible score can range from 0 to 100, with higher scores indicating greater diabetes-related emotional distress

### Prevalence of depression according to the different methods

**Fig. 1:**
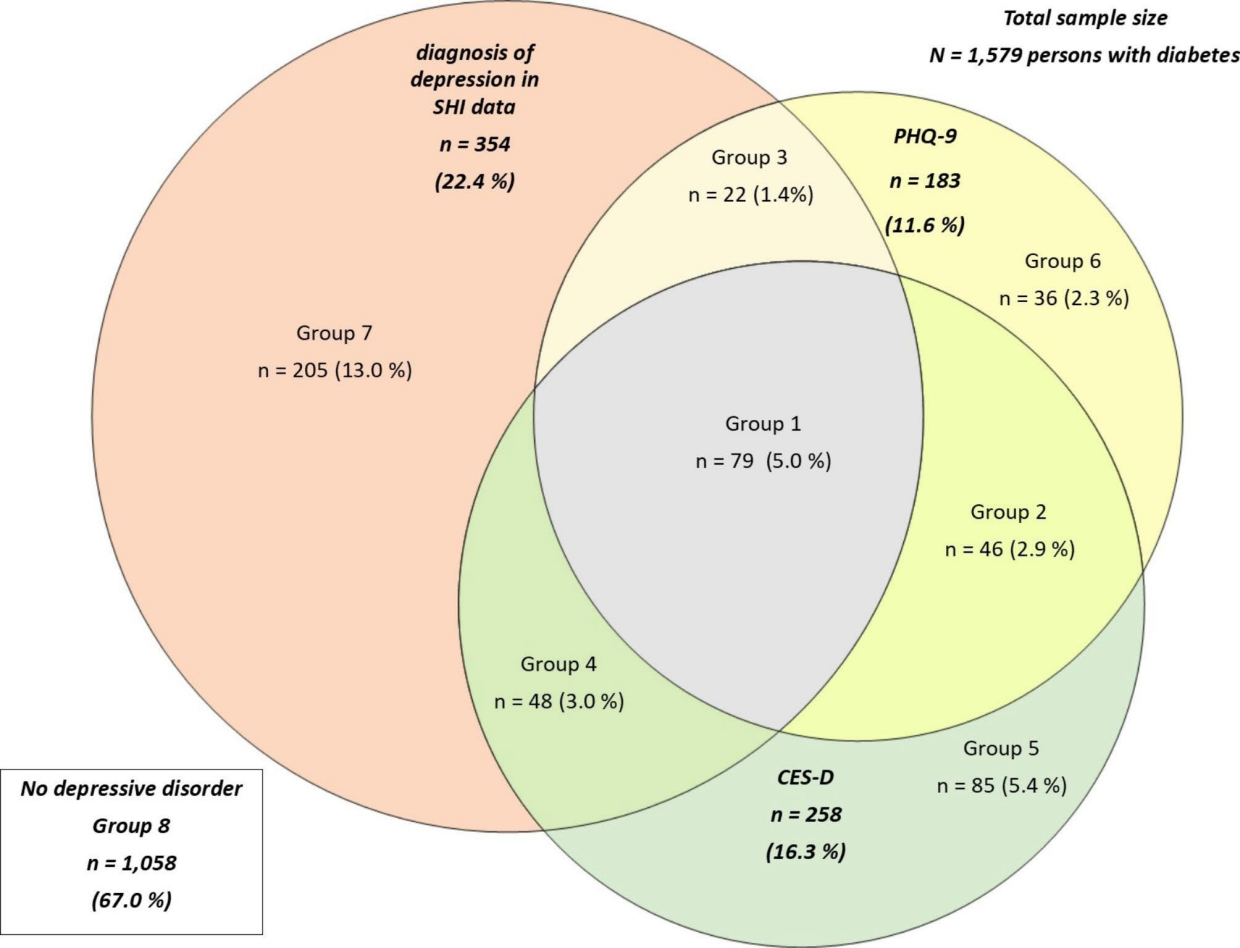
Venn diagram showing the persons identified by different methods to assess depressive disorder and intersections between the different methods

Figure 1 displays overlaps between the different methods and reports the overall prevalence within the sample. In total 33·0% of our analysis sample (521) were identified with some form of depression by at least one method. The prevalence of depression in our sample ranged from 11·6% (PHQ-9) up to 22·4% (SHI data).

The different groups and their characteristics are described in Table 2. Group 8 – the reference group - was the largest group with 1,058 persons and group 3 identified through the PHQ-9 and a diagnosis in SHI data the smallest with 22 persons. With respect to sociodemographic variables the percentage of females was highest in group 7 (51·7%) while it was lowest in group 6 (33·3%). Group 7 (only identified by a diagnosis in SHI data) was the group with most persons being German of origin (92·7%) and group 2 (identified by both instruments) the one with the smallest number of persons with German origin (76·1%). In group 8 most people were in a relationship (87·7%) and group 1 (identified by all methods) was the group where the smallest number of persons was in a relationship (70·1%). One third of group 6 (identified through PHQ-9 only) were retired but only about 52% of the persons in group 1. A duration of education for more than 14 years was highest in the group 8 (23·2%) and in group 1 (20·3%) and lowest in group 2 (15·2%). Group 1 had also the highest share of persons with type 1 diabetes (12·6%). With regard to diabetes specific and health care related outcomes, the highest number of persons with type 1 diabetes was found in group 1(12·8%) and the lowest amount was found in group 7 (4·9%). Group 2 and 3 had the highest share of persons taking insulin (43·5 and 50%) whereas in all other groups the share was around 30%. For OAD in all groups the share of persons taking them was between 60 and 70%. Average health care costs were highest in group 3 with a median of more than 13,900 € and lowest in group 8 (median 5,283 €).

**Table 2:** Comparison of groups according to depression status

	Group 1 n(%) / mean ± SD, median	Group 2 n(%) / mean ± SD, median	Group 3 n(%) / mean ± SD, median	Group 4 n(%) / mean ± SD, median	Group 5 n(%) / mean ± SD, median	Group 6 n(%) / mean ± SD, median	Group 7 n(%) / mean ± SD, median	Group 8 n(%) / mean ± SD, median	p-value
	***P + C + S+***	***P + C +*** S-	***P +*** C-***S+***	P-***C + S+***	P-***C +*** S-	***P +*** C-S-	P-C-***S+***	P-C-S-	
Sample size, n	79	46	22	48	85	36	205	1,058	
Age, years	61·3 ± 10·8, 62·0	64·9 ± 10·9, 65·0	62·4 ± 11·6, 61·0	63·7 ± 10·1, 65·0	66·5 ± 9·3, 68·0	69·7 ± 9·1, 72·5	67·2 ± 9·9, 69·0	67·7 ± 9·7, 70·0	**< 0·001**
Sex, female	38 (48·1)	20 (43·5)	8 (36·4)	24 (50·0)	33 (38·8)	12 (33·3)	106 (51·7)	356 (33·7)	**< 0·001**
Origin, Germany	63 (79·8)	35 (76·1)	19 (86·4)	37 (77·1)	73 (85·9)	28 (77·8)	190 (92·7)	952 (90·2)	**0·001**
Family status, in a relationship	54 (70·1)	35 (79·6)	17 (77·3)	38 (79·2)	62 (73·8)	29 (82·9)	155 (77·1)	916 (87·7)	**< 0·001**
Marital status									
Married	48 (60·8)	28 (60·9)	15 (68·2)	31 (64·6)	58 (69·1)	28 (77·8)	148 (72·6%)	832 (78·9%)	**< 0·001**
Divorce/separated	16 (20·3)	7 (15·2)	3 (13·6)	3 (6·3)	8 (9·5)	1 (2·8)	16 (7·8)	58 (5·5)	
Widowed	10 (12·7)	10 (21·7)	2 (9·1)	9 (18·8)	15 (17·9)	5 (13·9)	32 (15·7)	112 (10·6)	
Employment status, employed	20 (26·0)	16 (35·6)	5 (23·8)	16 (34·8)	25 (30·1)	8 (22·2)	44 (21·8)	268 (25·8)	0·44
Retirement status, retired	41 (51·9)	31 (67·4)	11 (50·0)	26 (54·2)	51 (61·5)	27 (75·0)	142 (69·6)	760 (72·7)	**< 0·001**
Level of education, ISCED ≥ 14 years	16 (20·3)	7 (15·2)	5 (22·7)	11 (22·9)	14 (16·7)	6 (16·7)	34 (16·7)	244 (23·2)	**0·05**
Diabetes duration, years	11·4 ± 8·9 8·5	11·8 ± 7·6 10	14·0 ± 10·4 10	12·7 ± 9·2 10	11·5 ± 8·0 10	11·2 ± 7·1 10	10·7 ± 8·8 8·5	10·9 ± 8·1 9	0·88
Type of Diabetes									
Type 1 Diabetes	10 (12·8)	3 (6·7)	3 (13·6)	6 (12·8)	8 (9·5)	2 (5·7)	10 (4·9)	86 (8·2)	0·07
Type 2 Diabetes	66 (84·6)	39 (86·7)	17 (77·3)	38 (80·9)	70 (83·3)	31 (88·6)	171 (83·8)	913 (86·9)	
Type unknown/other	2 (2·6)	3 (6·7)	2 (9·1)	3 (6·4)	6 (7·1)	2 (5·7)	23 (11·3)	52 (5·0)	
Diabetes severity aDCSI	2·9 ± 2·0, 3·0	3·3 ± 2·3, 3·0	3·2 ± 2·8, 2·5	3·6 ± 2·1, 3·0	3·0 ± 2·3, 2·0	4·1 ± 2·3, 4·0	3·6 ± 2·4, 3·0	2·9 ± 2·1, 3·0	**< 0·001**
Number of comorbidities	4·3 ± 2·0, 4·0	3·6 ± 1·6 3·0	4·6 ± 2·7 4·0	5·6 ± 2·8, 5·0	3·4 ± 1·9, 3·0	4·4 ± 2·5, 4·0	4·6 ± 2·5, 4·0	3·3 ± 1·9, 3·0	**< 0·001**
Treatment									
Taking insulin	27 (34·2)	20 (43·5)	11 (50·0)	17 (35·4)	29 (34·1)	12 (33·3)	57 (27·8)	313 (29·6)	0·17
Taking oral antihyperglycemic drugs	50 (63·3)	31 (67·4)	15 (68·2)	29 (60·4)	53 (62·4)	25 (69·4)	139 (67·8%)	729 (68·9)	0·81
Taking antidepressants	57 (72·2)	16 (34·8)	15 (68·2)	29 (60·4)	9 (10·6%)	4 (11·1)	89 (43·4)	57 (5·4)	**< 0·001**
Health care costs in € for 2 years	12,021·8 ± 9,631·2, 8,941·1	14,755·5 ± 22,196·0, 7,624·6	21,071·6 ± 23,499·2, 13,930·0	16,133·5 ± 16,576·6, 9,418·8	8,841·8 ± 8,848·2, 6,952·5	12,869·1 ± 12,829·9, 7,255·6	13,598·3 ± 15,942·2, 7,414·7	8,617·3 ± 11,730·2, 5,283·5	0·48
Health related Quality of Life									
physical component summary scale of the SF-12 (PCS-12)^$^	32·4 ± 9·3, 30·6	28·6 ± 7·6, 26·7	31·8 ± 10·2, 30·3	33·2 ± 9·3, 34·1	35·2 ± 9·6, 35·5	31·1 ± 7·8, 32·3	40·6 ± 10·5, 42·3	44·5 ± 9·8, 47·3	**< 0·001**
mental component summary scale of the SF-12 (MCS-12) ^$^	30·6 ± 7·8, 28·5	33·5 ± 6·8, 32·1	37·3 ± 9·1, 37·8	39·1 ± 7·7, 37·9	39·8 ± 7·6, 38·1	42·3 ± 7·3, 43·1	49·8 ± 10·2 52·2	54·1 ± 7·3, 56·0	**< 0·001**
Problem Areas in Diabetes Scale (PAID)^∞^	45·0 ± 22·5, 47·0	48·9 ± 19·8, 51·0	24·7 ± 20·3, 23·0	38·0 ± 19·7, 38·0	36·9 ± 16·7, 39·0	29·5 ± 18·4, 30·0	16·9 ± 13·5, 15·0	14·2 ± 12·7, 10·0	**< 0·001**
Previous depression									
Yes	53 (67·1)	15 (32·6)	11 (50·0)	28 (58·3)	4 (4·7)	2 (5·6)	60 (29·4)	52 (4·9)	**< 0·001**
No	8 (10·1)	13 (28·3)	5 (22·7)	9 (18·8)	42 (49·4)	22 (61·1)	98 (48·0)	844 (80·0)	
unknown	18 (22·8)	18 (39·1)	6 (27·3)	11 (22·9)	39 (45·9)	12 (33·3)	46 (22·6)	159 (15·1)	

SD = standard deviation

^$^ range from 0 to 100, zero indicates the lowest level of health measured by the scales and 100 indicates the highest level of health

^∞^ Possible score can range from 0 to100, with higher scores indicating greater diabetes-related emotional istress

PHQ-9, Patient Health Questionnaire-9; C = CES-D, Center for Epidemiological Studies Depression Scale; S = SHI data, statutory health insurance data

Group 1 = identified with all 3 instruments, Group 2 = identified by PHQ-9 and CES-D, Group 3 = identified by PHQ-9 and health insurance data, Group 4 = identified by CES-D and health insurance data, Group 5 = identified by CES-D, Group 6 = identified by PHQ-9, Group 7 = identified by health insurance data, Group 8 = no depressive disorder

Looking at HRQoL, the average score on the PCS12 was highest in group 8 (median 47·3) and group 7 (median 42·3) and lowest in group 1 (median 30·6). These findings were similar for the MCS12. The average PAID score was highest in group 1 (median 45·0) and lowest in group 8 (median 10·0) and group 7 (median 15·0). In group 1 was the highest share of persons reporting a previous depression (67·1%) and in group 8 the lowest share (4·9%).

### Results of the multinomial model

Table 3 reports the results of the multinomial logistic regression model with imputed data (the OOB imputation errors are reasonably small ranging from 0·086 to 0·71), comparing the seven groups with depressive disorder with the reference group with no depressive disorder (i.e., group 8). Overall, several differences in associations with the independent variables and the groups identified by the three methods were identified (even Bonferroni adjusted). We did not find a clear pattern between methods used and characteristics of individuals identified. However, we found some remarkable points.

First, we observed that a person who took antidepressants compared to a person who did not take antidepressants was 12 times (or for that matter about 9, 8 and 7 times) more likely to be in group 3 (group 1, 7 or 4, respectively) than in the reference group, i.e., OR = 12·00 (8·94, 8·31 and 7·25, respectively). These four groups are characterized by a diagnosis in SHI data. Contrastingly, in groups not identified through a diagnosis in SHI data, i.e., groups 2, 5 and 6, the estimated effects of taking antidepressants were considerably smaller and even not significant for groups 5 and 6 (depression symptoms according to CES-D and PHQ-9 only). A quite similar pattern was noticed for reporting previous depression and comorbidities: Persons reporting a previous depression where significantly more likely to be in one of the groups identified through a diagnosis in SHI data (group 1, 3, 4 and 7) compared to the reference group and people with more comorbidities were more likely to be groups 4 and 7 (both identified through SHI diagnosis).

Second, women were almost twice more likely to be in the group with an SHI data-based diagnosis only (group 7) than in the reference group (OR = 1.86). But there were no further significant associations related to other groups.

Third, age was a significant factor for group membership probability. With each year of life, it is less likely to be in any group with depressive disorder than in the reference group (all OR’s are less than one), however, not significant for groups without SHI-based depression diagnosis. Low HRQoL values and especially low MCS-12 values were associated with belonging to any group but not the one identified by SHI data only, each in comparison to the reference group. We observed that a person with low MCS-12 is significantly more likely to be in a group with both symptoms according to PHQ-9- and CES-D (i.e., group 1 and group 2) than in any other group. Furthermore, the results regarding the PAID Score point in the same direction, values were associated with belonging to any group (except the smallest group) but not the one identified by SHI data only.

**Table 3:** Results of the multinomial model reporting odds ratio (OR) and 95% confidence intervals (95% CI) for belonging to the different groups compared to belonging to the group with no depression (group 8)

	Group 1 (n = 79)	Group 2 (n = 46)	Group 3 (n = 22)	Group 4 (n = 48)	Group 5 (n = 85)	Group 6 (n = 36)	Group 7 (n = 205)
**Outcome**	***P + C + S+***	***P + C +*** S-	***P +*** C-***S+***	P-***C + S+***	P-***C +*** S-	***P +*** C-S-	P-C-***S+***
	OR^§^ [95% CI] (p-value)	OR^§^ [95% CI] (p-value)	OR^§^ [95% CI] (p-value)	OR^§^ [95% CI] (p-value)	OR^§^ [95% CI] (p-value)	OR^§^ [95% CI] (p-value)	OR^§^ [95% CI] (p-value)
**Age (years)**	0·94 [0·90, 0·98] **(0.0019)**	0·96 [0·92, 1·00] (0.0559)	0·93 [0·89, 0·98] **(0.0059)**	0·94 [0·91, 0·98] **(0·0024)**	0·97 [0·94, 1·01] (0·1025)	0·99 [0·95, 1·04] (0·7203)	0·98 [0·96, 1·00] **(0·0151)**
**Sex** **(female vs. male** ^**$**^ **)**	1·80 [0·86, 3·76] (0·1990)	1·09 [0·49, 2·54] (0·8317)	0·77 [0·28, 2·11] (0·6087)	1·91 [0·94, 3·91] (0·0756)	1·01 [0·57, 1·77] (0·9804)	0·67 [0·31, 1·45] (0·3084)	1·86 [1·31, 2·65] (0·0005*)
**Comorbidities (number)**	1·12 [0·94, 1·35] (0·2052)	0·82 [0·65, 1·03] (0·0924)	1·09 [0·87, 1·37] (0·4627)	1·44 [1·23, 1·68] (< 0·0001*)	0·91 [0·78, 1·06] (0·2183)	1·08 [0·90, 1·28] (0·4134)	1·31 [1·20, 1·44] **(< 0·001)**
**taking insulin** **(yes vs.no** ^**$**^ **)**	0·52 [0·24, 1·16] (0·1094)	0·86 [0·37, 1·97] (0·7157)	1·43 [0·52, 3·92] (0·4882)	0·48 [0·22, 1·06] (0·0678)	0·72 [0·40, 1·32] (0·2915)	0·58 [0·26, 1·29] (0·1814)	0·57 [0·37, 0·86] **(0·0075)**
**taking antidepressants** **(yes vs. no** ^**$**^ **)**	8·94 [3·87, 20·63] (< 0·0001*)	2·99 [1·16, 7·74] **(0·0235)**	12·00 [4·16, 34·65] (< 0·0001*)	7·25 [3·25, 16·15] (< 0·0001*)	1·24 [0·52, 2·93] (0·6244)	1·25 [0·40, 3·92] (0·7019)	8·31 [5·43, 12·73] (< 0·0001*)
**Health related Quality of Life**							
**physical component summary scale of the SF-12 (PCS-12 (score)**	0·90 [0·86, 0·95] (< 0·0001*)	0·84 [0·79, 0·89] (< 0·0001*)	0·89 [0·84, 0·94] **(0·0001)**	0·94 [0·90, 0·99] **(0·0106)**	0·94 [0·91, 0·97] (< 0·0001*)	0·90 [0·86, 0.94] (< 0·0001*)	0.99 [0.97, 1.01] (0·3628)
**mental component summary scale of the SF-12 (MCS-12) (score)**	0·73 [0·69, 0·78] (< 0·0001*)	0·75 [0·71, 0·80] (< 0·0001*)	0·86 [0·81, 0·91] (< 0·0001*)	0·88 [0·84, 0·92] (< 0·0001*)	0·85 [0·82, 0·88] (< 0·0001*)	0·88 [0·84, 0·92] (< 0·0001*)	0·98 [0·95, 1·00] **(0·0396)**
**Problem Areas in Diabetes Scale (PAID) (score)**	1·07 [1·05, 1·10] (< 0·0001*)	1·08 [1·06, 1·11] (< 0·0001*)	1·00 [0·97, 1·03] (0·8948)	1·06 [1·04, 1·08] (< 0·0001*)	1·06 [1·04, 1·08] (< 0·0001*)	1·03 [1·01, 1·06] **(0·0062)**	1·00 [0·98, 1·01] (0·7238)
**Previous depression**							
**(yes vs. no** ^**$**^ **)**	8·32 [2·83, 24·48] (< 0·0001*)	2·07 [0·66·6·42] (0·2099)	6·86 [1·86, 25·29] **(0·0038)**	9·11 [3·43, 24·20] (< 0·0001*)	0·37 [0·11, 1·23] (0·1041)	0·52 [0·11, 2·52] (0·4188)	5·19 [3·10, 8·68] (< 0·0001*)
**(Do not know vs. no** ^**$**^ **)**	2·76 [0·97, 7·85] (0·0569)	1·74 [0·68, 4·42] (0·2465)	2·47 [0·68, 8·96] (0·1691)	2·13 [0·80, 5·70] (0·1317)	1·49 [0·84, 2·66] (0·1752)	1·15 [0·52, 2·56] (0·7308)	1·73 [1·11, 2·71] **(0·0158)**

P = PHQ-9, Patient Health Questionnaire-9; C = CES-D, Center for Epidemiological Studies Depression Scale; S = SHI data, statutory health insurance data, MCS-12 = Mental summary score of the SF-12, PCS-12 = Physical summary score of the SF-12, PAID = Problem Areas in Diabetes scale

Group 1 = identified with all 3 methods, Group 2 = identified by PHQ-9 and CES-D, Group 3 = identified by PHQ-9 and health insurance data, Group 4 = identified by CES-D and health insurance data, Group 5 = identified by CES-D, Group 6 = identified by PHQ-9, Group 7 = identified by health insurance data, Group 8 = no depressive disorder

^**$**^Reference group, OR = odds ratio (corresponding to one unite change in case of age, comorbidities, PCS-12, MCS-12 and PAID)

CI = confidence interval, p-values under 0·05 are bold, *p-values significant at multiple level α/70

Nagelkerke’s R^2^ = 0.677

## Discussion

National and international guidelines recommend screening people with diabetes for depression to identify patients in need of psychological treatment [31, 32]. However, neither of these guidelines give detailed instructions on which screening instrument to use or describe the differences for the identified groups. A recent meta-analysis of diagnostic accuracy of depression questionnaires in adult patients with diabetes by de Joode et al. (2019) showed, that the CES-D and the PHQ-9 are the most frequently evaluated depression questionnaires among patients with diabetes [20]. They differed in terms of sensitivity and specificity, however none of the two instruments was found to be superior over the other. The results of our study show that between 14·6% and 22·4% of individuals with diabetes had depression depending on the method used to assess it. High prevalence estimates can be expected, since on the one hand, there is evidence that depression is a risk factor for diabetes and, on the other hand, studies show that the distress caused by diabetes contributes to the development of depression [1, 8, 33–36]. The results of our study are within the range of findings from the two most recent meta-analyses on depression among persons with diabetes where prevalence ranged from 1·8% up to 88·0% [1, 8]. One could assume, that both instruments used would identify more or less the same persons since they both measure depression symptoms within the last or the last two weeks. One could also assume an overlap between the two instruments and the persons identified through SHI data, however this overlap would be expected to be a little less pronounced as SHI data covers diagnosis from two years. We indeed found some overlap between the methods; however, surprisingly the majority of persons was identified by one instrument only (20·7% of the total sample), 7·3% of the whole sample were identified using two methods and 5·6% were identified with all three methods. The largest number of persons was identified through SHI data only (group 7). In total 68·0% of those identified with depression in our sample were identified through SHI data of which 42·1% were also identified through one of the two instruments. The characteristics of individuals identified by either of the two instruments were quite similar. Within our sample women were more likely to belong to the group identified through SHI data only (group 7). This is in line with results of an analysis of routine German SHI data that found women are diagnosed more frequently than men in all age groups [37].

It seems that persons who have a diagnosis of depression in SHI data but do not show symptoms in either of the questionnaires (group 7) do not noticeably differ in their HRQoL when compared with the group with no depression. Neither were the reported scores for diabetes related distress high in this group.

Screening for depression among individuals with diabetes seems to be necessary since all groups identified through at least one questionnaire (groups 1–7) had more unfavorable outcomes compared to the group with no depression.

Our findings show that, even though the same disease should be measured, the degree of variability in persons identified across the methods is substantial. If we would have used the PHQ-9 only we would have missed 133 patients who have depressive symptoms according to the CES-D but not according to the PHQ-9. Similarly, if we would have used only the CES-D we would have missed 58 persons who had symptoms according to the PHQ-9 but not according to the CES-D. Unfortunately, the differences between the groups were not pronounced enough to draw conclusions on which method is to be preferred.

To keep in mind: We found some indication that the method chosen to identify persons with a depressive disorder might be related to particular underlying characteristics in the population identified. To our knowledge, there is no study, which has used a similar many-to-one approach. It will be interesting to compare findings of future studies with larger samples.

### Strength and limitations

To our knowledge, this is the first study which analyses groups identified by different instruments to assess depression, and includes also SHI data. The linkage of survey data with SHI data allows a more detailed description of the identified persons which would not be possible with using only one of the two data sources. The analyzed data set is rather large allowing for robust estimates. Moreover, the response rate was with 51·0% reasonably high for a survey-based study. An nonresponse analysis did not reveal any major differences between responders and nonresponders especially with respect to depression [23]. Thus, nonresponse bias should be small. However only persons of one SHI could participate in the study which might influence the results since, for example, the prevalence of diabetes varies among the different SHIs in Germany [38]. Survey data was only assessed at one point during the study period whereas the SHI data covers the whole study period thus the prevalence observed in SHI data might be, among other reasons, higher as the time frame during which it is assessed is longer. Moreover, it has to be kept in mind that a diagnosis in SHI data is not valid as a screening measure for depression since people with a diagnosis have most likely received some form of therapy. Furthermore, within SHI data we find clinical diagnosis whereas the results of the CES-D and the PHQ-9 are not clinical diagnosis but results of screening measures for depression. Additionally, it might be the case that once a person has received a depression diagnosis it will not be removed from the track record even though the person does not have depression anymore. Likewise, we could not get a full history of diagnosed depression but only data on depression diagnosis 12 month before and after the baseline assessment. Our focus is on acute depression, in line with the two instruments used during the baseline survey, which is not covered by a lifetime history of depression. Since we include a considerable time frame before and after the baseline assessment misclassification is assumed to be low.

## Conclusion

Our study is the first study that describes the overlap and differences between individuals identified with different methods to detect depression. Although several characteristics were found to be associated with belonging to the different groups; we did not find a clear pattern among those characteristics. However, we have found some initial indications that the method chosen is related to particular underlying characteristics in the population identified. The methods have a relatively low overlap. The majority of persons were identified using a diagnosis in SHI data. Those identified through SHI data only did not differ in their HRQoL when compared to those with no depression. This could be either due to a successful therapy or due to a spontaneous relapse. Our study shows, that there might be similarities but also differences in characteristics of identified persons depending on the method used. By using either of the three methods, one should be aware that certain persons are missed. Therefore, further research with a comprehensive data set, that is sufficiently large in terms of case numbers, is needed to address the implications of using either of the methods.Especially prospective studies investigating clinical outcomes would be important. This knowledge is crucial to enable clinicians to make an informed decision about the usage of either of the two instruments in every day practice, taking into account setting, time constraints and other relevant circumstances.

## Electronic supplementary material

Below is the link to the electronic supplementary material.

Supplementary Material 1

Supplementary Material 2

Supplementary Material 3

## Data Availability

Due to ethical concerns, supporting data on the results of the questionnaire cannot be made openly available. Additionally, data of the statutory health insurance was already existing and was obtained upon request and subject to licence restrictions from a number of different sources. Full details how these data were obtained are available in the documentation available at: 10.14312/2398-0281.2016-3.

## Declarations

### Statement to confirm that all methods were carried out in accordance with relevant guidelines and regulations

We have adhered to best practice guidelines for Strengthening the Reporting of Observational Studies in Epidemiology (STROBE).

### Precis

Different instruments to screen for depression do not identify the same persons, however no systematic differences exist between the different groups of persons.
