# Supplementary figures and images for: PHQ-9, CES-D, health insurance data—who is identified with depression? A Population-based study in persons with diabetes
Source: Diabetol Metab Syndr. 2023 Mar 22;15:54. doi: 10.1186/s13098-023-01028-7 (PMC10031874; doi:10.1186/s13098-023-01028-7)

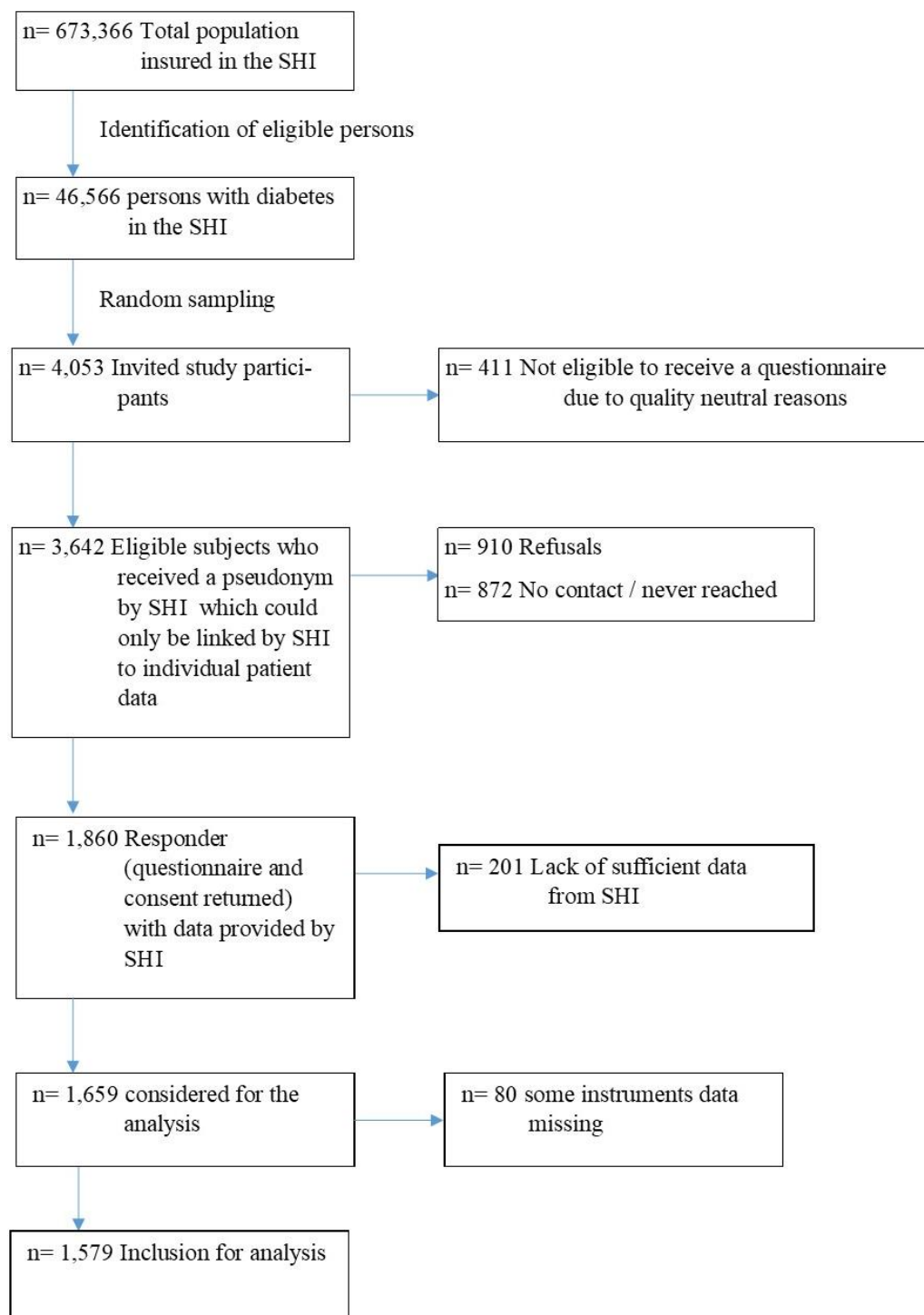

Appendix Figure 1: Flowchart of study participants

Supplement: Supplementary file 1 — Supplementary Material 1 [file 13098_2023_1028_MOESM1_ESM.pdf]
