# Supplementary material for: PHQ-9, CES-D, health insurance data—who is identified with depression? A Population-based study in persons with diabetes
Source: Diabetol Metab Syndr. 2023 Mar 22;15:54. doi: 10.1186/s13098-023-01028-7 (PMC10031874; doi:10.1186/s13098-023-01028-7)
Supplement: Supplementary file 2 — Supplementary Material 2 [file 13098_2023_1028_MOESM2_ESM.pdf]

	PHQ-9* **P**	CES-D# **C**	SHI data$ **S**
Group 1	+	+	+
Group 2	+	+	-
Group 3	+	-	+
Group 4	-	+	+
Group 5	-	+	-
Group 6	+	-	-
Group 7	-	-	+
Group 8	-	-	-

* PHQ-9, Patient Health Questionnaire-9
# CES-D, Center for Epidemiological Studies Depression Scale
$ SHI data, statutory health insurance data
+ identified as having depressive disorder through this instrument
– not identified as having depressive disorder through this instrument

Appendix table 1: Group composition based on depression measurement
