# Supplementary material for: PHQ-9, CES-D, health insurance data—who is identified with depression? A Population-based study in persons with diabetes
Source: Diabetol Metab Syndr. 2023 Mar 22;15:54. doi: 10.1186/s13098-023-01028-7 (PMC10031874; doi:10.1186/s13098-023-01028-7)
Supplement: Supplementary file 3 — Supplementary Material 3 [file 13098_2023_1028_MOESM3_ESM.pdf]

	PHQ-9[+]			CES-D[#]			SHI data[$]		
	No depression symptoms	depression symptoms	**p-value**	No depression symptoms	depression symptoms	**p-value**	No depression symptoms	depression diagnosis	**p-value**
Sample size, n	1396	183		1321	258		1225	354	
Age (mean ± SD), years<br>Median	67.4 ± 9.7<br>70.0	64.0 ± 11.0<br>64.0	0.001**	67.6 ± 9.7<br>70.0	64.1 ± 10.4<br>64.0	0.001**	67.5 ± 9.7<br>70.0	65.1 ± 10.5<br>66.0	0.001**
Sex, female n (%)	519 (37.2 %)	78 (42.6 %)	0.18	482 (36.5 %)	115 (44.6 %)	0.005**	421 (34.4 %)	176 (49.7 %)	0.001**
Origin, Germany n (%)	1252 (89.8 %)	145 (79.2 %)	0.001**	1189 (90.1 %)	208 (80.6 %)	0.001**	1088 (89.0 %)	309 (87.3 %)	0.44
Family status, in a relationship, n (%)	1171 (85.0 %)	135 (75.8 %)	0.001**	1117 (85.7 %)	189 (74.7 %)	0.001**	1042 (86.3 %)	264 (75.9 %)	0.001**
Marital status, n (%)									
Married	1069 (76.9 %)	119 (65.0 %)	0.001**	1023 (77.7 %)	165 (64.2 %)		946 (77.5 %)	242 (68.6 %)	0.001**
Divorce/separated	85 (6.1 %)	27 (14.8 %)		78 (5.9 %)	34 (13.2 %)	0.001**	74 (6.1 %)	38 (10.8 %)	
Widowed	168 (12.1 %)	27 (14.8 %)		151 (11.5 %)	44 (17.1 %)		160 (11.9 %)	35 (15.2 %)	
Employment status, employed n (%)	353 (25.8 %)	49 (27.4 %)	0.72	325 (25.1 %)	77 (30.7 %)	0.08	142 (11.6 %)	53 (15.0 %)	0.54
Retirement status, retired n (%)	979 (70.9 %)	110 (60.1 %)	0.001**	940 (71.9 %)	149 (58.2 %)	0.001**	869 (71.8 %)	220 (62.3 %)	0.001**
Level of education, ISCED ≥14 years, n (%)	303 (21.9 %)	34 (18.6 %)	0.005**	289 (22.0 %)	48 (18.7 %)	0.001**	271 (22.3 %)	66 (18.7%)	0.001**
Diabetes duration (mean ± SD), years<br>median	10.9 (8.3)<br>9.0	11.8 (8.5)<br>10.0	0.64	10.9 (8.3)<br>9.0	11.7 (8.4)<br>10.0	0.5	11.0 ± 8.3<br>9.0	11.0 (8.1)<br>9.0	0.36
Type of Diabetes, n (%)									
Type 1 Diabetes	110 (7.9 %)	18 (10.0 %)	0.65	101 (7.7 %)	27 (10.6 %)	0.36	99 (8.3 %)	29 (8.3 %)	0.10
Type 2 Diabetes	1192 (86.0 %)	153 (85.0 %)		1132 (86.3 %)	213 (83.9 %)		1053 (86.7 %)	292 (83.2 %)	
Type unknown/other	84 (6.1 %)	9 (5.0 %)		79(6.0 %)	14 (5.5 %)		63 (5.2 %)	30 (8.5 %)	
Diabetes severity aDCSI (mean ± SD)<br>median	3.0 (2.2)<br>3.0	3.3 (2.3)<br>3.0	0.39	3.0 (2.2)<br>3.0	3.1 (2.2)<br>3.0	0.005**	2.9 (2.3)<br>3.0	3.4 (2.3)<br>3.0	0.05
Number of comorbidities (mean ± SD)<br>median	3.6 (2.1)<br>3.0	4.2 (2.1)<br>4.0	0.001**	3.6 (2.1)<br>3.0	4.1 (2.2)<br>4.0	0.001**	3.4 (1.9)1384<br>3.0	4.7 (2.5)<br>4.0	<0.001**
Treatment, n (%)									
Taking insulin	416 (29.8 %)	70 (38.3 %)	0.001**	393 (29.8 %)	93 (36.1 %)	0.05	374 (30.5 %)	112 (31.6 %)	0.74
Taking oral antihyperglycemic drugs	950 (68.1 %)	121 (66.1 %)	0.66	908 (68.7 %)	163 (63.2 %)	0.09	838 (68.4 %)	233 (65.8 %)	0.39
Taking antidepressants	184 (13.2 %)	92 (50.3 %)	<0.001**	165 (12.5 %)	111 (43.0 %)	<0.001**	86 (7.0 %)	190 (53.7 %)	<0.001**
Health care costs for 2 years (mean ± SD), €<br>median	9619.5<br>(12648.0)<br>5828.1	13963.6<br>(16281.2)<br>8627.5	0.49	9712.1<br>(12974.2)<br>5794.8	12226.5.1<br>(14073.2)<br>7773.7	0.49	8988.3 (12199.6)<br>5495.7	14049.5<br>(15540.8)<br>8187.7	0.49
Health related Quality of Life (mean ± SD)<br>median									
PCS12	43.0 (10.4)<br>44.7	31.1 (8.8)<br>29.0	<0.001**	43.4 (10.3)<br>45.7	32.8 (9.3)<br>31.8	0.005**	43.0 (10.6)<br>44.8	37.3 (10.8)<br>36.4	<0.001**
MCS12	52.1 (8.9)<br>55.1	34.2 (8.7)<br>32.7	<0.001**	52.9 (8.4)<br>55.6	35.8 (8.6)<br>34.9	0.001**	52.0 (9.0)<br>55.2	43.4 (12.3)<br>42.3	<0.001**
PAID	16.8 (15.0)<br>13.0	40.6 (22.5)<br>41.0	0.001**	15.2 (13.4)<br>11.0	41.5 (20.1)<br>43.0	<0.001**	17.4 (16.0)<br>13	26.3 (21.0)<br>23	0.001**
Previous depression, n (%)									
Yes	144 (10.3 %)	81 (44.3 %)	<0.001**	125 (9.5 %)	100 (38.7 %)	0.001**	73 (6.0 %)	152 (43.1 %)	<0.001**
No	993 (71.3 %)	48 (26.2 %)		969 (73.6 %)	72 (27.9 %)		921 (75.4 %)	120 (34.0 %)	
unknown	255 (18.3 %)	54 (29.5 %)		223 (16.9 %)	86 (33.3 %)		228 (18.7 %)	81 (23.0 %)	

+ PHQ-9, Patient Health Questionnaire-9
# CES-D, Center for Epidemiological Studies Depression Scale
$ SHI data, statutory health insurance data
** - the difference between groups were statistically significant.

Appendix table 2: Description of persons identified by the different methods.
